# Assessing readiness for evidence-based practice among healthcare professionals in Egypt

**DOI:** 10.1186/s12909-025-07401-6

**Published:** 2025-06-01

**Authors:** Ghaidaa A. Ahmed, Maggie M. Abbassi, Samar F. Farid, Nirmeen A. Sabry

**Affiliations:** 1 Department of Clinical Pharmacy, College of Pharmaceutical Science and Drug Manufacturing, Misr University for Science and Technology, Giza, Egypt; 2 Department of Clinical Pharmacy, Faculty of Pharmacy, Cairo University, Kasr El-Aini St, P.O. Box: 11562, Cairo, Egypt

**Keywords:** Evidence-based medicine, Evidence-based practice, Health care professionals, Perception, Attitude, Practice

## Abstract

**Background:**

Evidence-Based Practice (EBP) implements individual clinical experience along with the highest accessible clinical evidence. Nowadays, EBP is an essential part of decision-making in many healthcare fields including nursing, pharmacy, and physiotherapy to improve patient care and healthcare outcomes. Literature showed many barriers and considerable gaps between the available scientific evidence and the services provided by healthcare professionals (HCPs). This study aimed to assess the awareness of, attitudes toward, barriers to, and implementation of evidence-based practice among Egyptian healthcare professionals.

**Methods:**

A cross-sectional observational study was conducted from April 2023 and February 2024 using an online SurveyMonkey questionnaire targeting physicians, pharmacists, and nurses in primary and secondary care settings in Egypt. The questionnaire was designed based on a literature review, adapted to cultural relevance, and validated to explore awareness, attitudes, practices, and barriers related to EBP.

**Results:**

Among 1396 participants, significant demographic differences were observed. Most pharmacists and nurses were females. Nurses were the youngest while physicians were the most experienced. Most HCPs were aware of EBP and showed positive attitudes towards EBP. However, basic EBP terms and EBP use in decision-making were mostly utilized by physicians followed by pharmacists, while nurses were the least to implement EBP. The most commonly reported barriers were the lack of EBP training courses, the inaccessibility to full-text academic journals due to high cost, and the cost of implementing new treatments in Egypt.

**Conclusion:**

While Egyptian healthcare professionals generally supported the principles of evidence-based practice and recognized its impact in improving patient outcomes, barriers such as limited time, high costs, and restricted access to resources hinder the effective implementation of EBP in Egypt. Positive attitude towards EBP was observed mostly in physicians and pharmacists and to a lesser extent in nurses. Future initiatives should focus on increasing access to EBP resources, integrating EBP training into undergraduate education curricula, and providing support for continuous professional development in evidence-based practices.

## Introduction

Evidence-based medicine (EBM) is defined as “the conscientious, explicit, and judicious use of current best evidence in making decisions about the care of individual patients” [1]. This approach integrates clinical expertise and patient’s values with the best available external clinical evidence from systematic research in the process of decision making related to patients’ health care [2]. EBM was presented to shift from information relying on expert opinion to more superior quality evidence based on clinical studies [3]. Additionally, the concept introduced in 1991 was initiated to help healthcare professionals (HCP) criticize accessible research evidence and customize it to specific patients and circumstances [4]. The practice of classifying “levels of evidence” which ranks clinical studies based on the strength and reliability of their findings to customize treatment for individual cases is a fundamental aspect of evidence-based medicine (EBM) [5].Now, EBM is practiced across all healthcare professions (HCPs), including medicine, pharmacy, nursing, physiotherapy, and allied health fields, with varying levels of awareness, knowledge, and implementation among these groups [6].

Although a large portion of the world’s diseases and infections are found in low-income nations, the majority of these countries suffer from deficient public health systems [7, 8] Moreover, scarce literature regarding EBM applications in Africa has been detected compared to that in developed nations, as highlighted in discussions on strengthening evidence-based healthcare in Africa and its relevance to public health in developing countries [9, 10].

Furthermore, published studies regarding the middle and low-income countries revealed numerous barriers that could limit the benefits of EBM. For example, the lack of research skills comprising inadequate training, inadequate funding or grants, a lack of encouraging research-friendly government or institutional policies [11], and a lack of research facilities in these countries has adversely affected healthcare quality [12].

In Egypt, EBP necessitates a lot of effort to ensure the establishment and implementation of the technique in diverse health fields and identify specific barriers to its adoption. One study examined the knowledge and EBP among physicians at the National Liver Institute, Menoufia University revealed a variation of knowledge among the physicians highlighting lack of time, lack of clinic facilities, patient beliefs and preferences as barriers to EBP in Egypt [13]. Another study revealed an absence of EBP among nurses due to a deficiency of knowledge and structured training introduced to nurses during their undergraduate study or their career life [14]. These studies spotlight the variability of the knowledge, attitude, and practice (KAP) in EBP among different HCPs.

Accordingly, this study aimed to assess Egyptian HCPs’ KAP of EBP. In addition, the present study aimed to mention the barriers that could hold or prevent professionals from EBP and how EBP can be developed and expanded in Egypt.

## Methods

### Study design

This descriptive cross-sectional, questionnaire-based study was conducted using SurveyMonkey targeting HCPs in primary and secondary care settings between April 2023 and February 2024.

### Study population and sample size

The targeted population of the study was physicians, pharmacists, and nurses.

According to a report from the Egyptian syndicates of the three HCPs (November 2023), there were 339,870 physicians, 315,000 pharmacists, and 25,0000 nurses in Egypt. Considering a margin of error and confidence interval of 5% and 95%, respectively, a minimum sample size of 384 for each HCP category was required. The sample size was calculated using SurveyMonkey online sample size calculator.

### Ethics approval and consent to participate

The study protocol was approved by the Research Ethics Committee for Experimental and Clinical Studies at the Faculty of Pharmacy, Cairo University (Approval Number: CL(2252)), effective since July 2018. The committee reviewed the study in accordance with institutional and international ethical guidelines.

The committee waived the requirement for explicit written informed consent, as the study utilized an anonymous survey and posed minimal risk to participants. Instead, an introductory statement was presented alongside the survey link, informing participants about the study’s purpose, its relevance to healthcare professionals in Egypt, and the estimated completion time (10–15 min). The statement also assured participants that their responses would remain anonymous, confidential, and used solely for research purposes. Completion of the survey was considered implied consent.

This study was conducted in full compliance with the ethical principles outlined in the Declaration of Helsinki (https://www.wma.net/policies-post/wma-declaration-of-helsinki/).

### Questionnaire development and validation

#### Item construction and selection

The questionnaire was developed based on a systematic review of validated instruments used in previous research assessing knowledge, attitudes, and practices (KAP) regarding evidence-based practice (EBP) among healthcare professionals [15–19]. Items were selected to align with the core EBP domains: awareness and training, attitudes toward EBP, and reported engagement and barriers. The selection aimed to ensure content coverage, contextual relevance to the Egyptian healthcare system, and alignment with current evidence-based competencies.

#### Face and content validation

To ensure both face and content validity, the draft questionnaire was reviewed by five senior teaching assistants from the Faculty of Pharmacy, Cairo University, who had expertise in EBP and clinical research. They evaluated the clarity, relevance, and appropriateness of each item. Their feedback led to minor revisions for improved clarity and contextual alignment.

The questionnaire was originally drafted in English and then professionally translated into Arabic by a certified translation institute. A bilingual subject-matter expert conducted a back-translation to ensure linguistic and conceptual equivalence between the two versions.

The instrument was piloted with a diverse sample of 22 healthcare professionals (4 physicians, 14 pharmacists, 4 nurses) representative of the target population. Participants completed the survey via an online platform under conditions identical to the actual study. The investigator was present during completion to collect real-time feedback on question clarity, item relevance, ambiguity, double meaning questions and technical issues. Minor adjustments were made based on this feedback, including rewording for clarity and reordering some items for better logical flow. The average completion time was between 22 and 30 min.

The final questionnaire consisted of four structured sections totaling 44 items:

- Section I: Demographic and professional characteristics (11 items): age, sex, professional role, qualifications, workplace affiliation, and years of experience.
- Section II: Knowledge and awareness of EBP (6 items): This section used a combination of Yes/No, multiple-choice, and open-ended items to assess familiarity with the term “Evidence-Based Medicine,” prior EBP training, and understanding of basic EBP concepts such as literature search, question formulation, biostatistics, and critical appraisal.
- Section III: Attitudes toward EBP (17 items): Items were rated on a 5-point Likert scale ranging from 1 (“strongly disagree”) to 5 (“strongly agree”). The items addressed various aspects of EBP, including its integration into clinical practice, impact on patient outcomes, and value as an educational tool. To provide a clear and meaningful visualization of the data, the distribution of responses for each item has been presented using a bar chart. This approach allows for a more accurate depiction of the frequency of each response category and facilitates better interpretation of participants’ attitudes toward EBP.
- Section IV: EBP Practice and Implementation Barriers (10 items):
- This section captures healthcare professionals’ self-reported engagement in EBP-related activities, perceived barriers (such as lack of time or institutional support), and their views on factors that would facilitate the adoption of EBP. The responses were recorded as frequencies and percentages, and comparisons between groups were conducted using the chi-square test.

While no aggregate scoring was applied across all sections, individual item responses were analyzed descriptively. In Section III, the distribution of responses for each item was presented using bar charts to illustrate the frequency of agreement levels, providing a clearer picture of participants’ attitudes toward EBP. For Section IV, a chi-square test was used to compare practices among the three professions.

#### Reliability assessment

Reliability was assessed by calculating Cronbach’s alpha for each table in the survey. The results were coded, and Cronbach’s alpha values greater than 0.70 were observed across all tables, indicating strong internal reliability and acceptable consistency for survey-based research.

### Data collection

The English/Arabic language versions were distributed online by sharing the link along with an introductory statement across professional groups on various platforms such as Facebook and WhatsApp, professional networks, and institutional emails.

### Statistical analysis

Statistical analysis was conducted using the Statistical Package for Social Sciences (SPSS), version 25. Categorical variables were analyzed using the Chi-square test to compare responses among the three professional groups (physicians, pharmacists, and nurses). When expected cell counts were less than 5, Fisher’s Exact test was applied. Statistical significance was defined as a p-value < 0.05. Results were presented using bar charts generated with Microsoft Excel 2010 to visually summarize key comparisons and trends. Data collected during the preliminary (pilot) phase of the questionnaire were excluded from the final analysis.

## Results

A total of 1873 respondents participated in the survey. Respondents including non-university graduated nurses, students, academia, industry, dentists, veterinarians, and those working in sales and marketing were excluded from the study. Respondents belonging to research institutes such as National Research Center, the National Food Safety Authority, and the Egyptian Drug Authority were also excluded. A total of 1667 respondents were left, and respondents who filled in demographic data only were removed. Finally, 1396 were included in the analysis.

According to Table 1, several significant demographic differences among pharmacists, nurses, and physicians emerged. A statistical significance was observed among the three groups (pharmacists, nurses, and physicians) for each variable. Pharmacists who responded to the questionnaire predominantly consisted of females, while physicians had the lowest percentage of female representation among the three groups. Nurses were generally younger in age on average compared to pharmacists and physicians, reflecting potential differences in career stage and experience. A substantial majority across all professions graduated from public universities, and nurses showed the highest proportion. Physicians tended to have more years of experience on average compared to pharmacists and nurses. There were varied levels of postgraduate education across the professions, with pharmacists and physicians showing higher proportions in advanced degrees like MSc and Ph.D/MD.

**Table 1:** Respondents’ characteristics are presented as mean, standard deviation, frequency and percentage

Variable	Pharmacists *N* = 672	Nurses *N* = 296	Physicians *N* = 428
Gender, Female, n (%)	531 (79.02)	159 (53.72)	196 (45.80)
Age (Mean+/-SD)	32.21 ± 8.07	30.967 ± 9.03	35.43 ± 9.57
University of Graduation Public, n (%)	431 (64.14)	253 (85.47)	352 (82.24)
Years of Experience (Mean+/-SD)	8.57 ± 5.49	7.36 ± 3.38	9.36 ± 6.41
Medical Postgraduate Studies			
No post-graduate studies, n (%)	404 (60.12)	213 (71.96)	154 (35.98)
Diploma, n (%)	146 (21.73)	26 (8.78)	38 (8.88)
Pharm D, n (%)	46 (6.85)	-	-
MSc, n (%)	41 (6.10)	38 (12.84)	137 (32.01)
Ph.D./M.D., n (%)	6 (0.89)	15 (5.07)	66 (15.42)
Others, n (%)	29 (4.31)	4 (1.35)	33 (7.71)

Significance difference, *Chi-squared test and #ANOVA were used *Fisher’s Exact Test was done for cells count less than 5.

Table 2 presents data on HCPs’ awareness of EBM and their participation in related courses or workshops. The study findings indicated varying levels of awareness and engagement with EBP among healthcare professionals. While most participants were aware of EBM, fewer have actively participated in courses or workshops on the subject. There was also a notable gap in training across specific EBP skills, with a significant portion of professionals indicating they have not received training in question formulation, critical appraisal, or biostatistics.

Although not all healthcare practitioners in Egypt are directly involved in scientific research, competencies related to evidence-based practice (EBP)—such as formulating clinical questions, conducting literature searches, and applying basic biostatistics—are increasingly being integrated into clinical decision-making and professional development initiatives. This trend is particularly evident in university hospitals and tertiary care centers, where healthcare professionals are encouraged to adopt evidence-based protocols and engage in quality improvement efforts. National initiatives and institutional programs, such as those led by the Oxford–Egypt Evidence-Based Healthcare Alliance and the Evidence-Based Healthcare Program at Children’s Cancer Hospital Egypt (CCHE) 57,357, have played a significant role in promoting EBP education and practice among clinicians across the country [20, 21]. Studies conducted in institutions like Tanta University Hospitals and the Egyptian Liver Research Institute further support the growing emphasis on EBP by highlighting the positive impact of EBP training on clinical practice and organizational culture [22, 23] Moreover, broader assessments of continuing professional development in Egypt underscore the importance of embedding EBP principles in healthcare training and practice [24, 25].

**Table 2:** Participants’ responses to questions about awareness of EBM and related courses are represented as frequency and percentage

Parameter	Options		Profession		*P* value
		Pharmacists *N* = 672	Nurses *N* = 296	Physicians *N* = 428	
Knowledge about the term “Evidence-Based Medicine” (EBM)?	Yes, n (%)	382 (56.85)	198 (66.89)	348 (81.31)	0.00*
Have you ever attended a course or workshop on EBM?	Yes, n (%)	116 (17.26)	57 (19.26)	106 (24.77)	0.010*
Receiving training in any of the following fields?	Question formulation, n (%)	60 (8.99)	46 (15.54)	40 (9.35)	0.026*
	Literature search/Research methodology, n (%)	135 (20.09)	82 (27.70)	143 (33.41)	0.00*
	Critical appraisal, n (%)	59 (8.78)	39 (13.18)	35 (8.18)	0.077
	Biostatistics, n (%)	149 (22.17)	71 (23.99)	141 (32.94)	0.00*
	Not receiving any of these, n (%)	459 (68.30)	169 (57.09)	210 (49.07)	0.00*

*Significance difference, Chi square test is used

Table 3 outlines the level of understanding of some basic EBM terms among HCPs. For the term “relative risk and absolute risk”, physicians showed a significantly higher level of understanding compared to both nurses (*p* = 0.021) and pharmacists (*p* < 0.001), with pharmacists also outperforming nurses (*p* < 0.001). Similarly, for “randomized controlled trials” (RCTs), physicians demonstrated greater understanding than nurses (*p* < 0.001) and pharmacists (*p* < 0.001), while pharmacists were significantly better than nurses (*p* = 0.036).

When evaluating the term “meta-analysis”, significant differences were found, with physicians showing a higher understanding compared to both nurses (*p* < 0.001) and pharmacists (*p* < 0.001), and pharmacists also surpass nurses (*p* = 0.007). However, for “confidence intervals”, no significant differences in understanding were observed between pharmacists, nurses, and physicians (*p* > 0.05).

In terms of “publication bias”, physicians had a significantly higher understanding compared to nurses (*p* = 0.038). Pharmacists also demonstrated a greater understanding than nurses (*p* = 0.043). No significant difference was noted between pharmacists and physicians for this term (*p* = 0.112). These findings highlight the variation in EBP knowledge across the different healthcare professions, with physicians consistently demonstrating the highest level of understanding for most terms.

**Table 3:** Level of Understanding of basic EBM terms among health care professionals expressed in frequency and percentages

Term	Profession	I don’t understand and I’m not willing to.	I don’t understand but I would like to	I understand to some extent	I understand and I can explain to others	*P* value
Relative risk and absolute risk	Pharmacists, n (%)	53 (7.9)	232 (34.5)	296 (44)	91 (13.5)	0.00
	Nurses, n (%)	34 (11.5)	121 (40.9)	113 (38.2)	28 (9.5)	
	Physicians, n (%)	27 (6.3)	87 (20.3)	229 (53.5)	85 (19.9)	
Randomized controlled trials	Pharmacists, n (%)	59 (8.8)	201 (29.9)	242 (36)	170 (25.3)	0.00
	Nurses, n (%)	26 (8.8)	107 (36.1)	112 (37.8)	51 (17.2)	
	Physicians, n (%)	23 (5.4)	80 (18.7)	223 (52.1)	102 (23.8)	
Meta-analysis	Pharmacists, n (%)	73 (10.9)	315 (46.9)	179 (26.6)	105 (15.6)	0.00
	Nurses, n (%)	27 (9.1)	163 (55.1)	82 (27.7)	24 (8.1)	
	Physicians, n (%)	29 (6.8)	171 (40)	166 (38.8)	62 (14.5)	
Confidence interval	Pharmacists, n (%)	72 (10.7)	354 (52.7)	155 (23.1)	91 (13.5)	0.180
	Nurses, n (%)	25 (8.4)	155 (52.4)	58 (28.7)	31 (10.5)	
	Physicians, n (%)	33 (7.7)	236 (55.1)	114 (26.6)	45 (10.5)	
Publication bias	Pharmacists, n (%)	75 (11.2)	259 (38.5)	209 (31.1)	129 (19.2)	0.020
	Nurses, n (%)	30 (10.1)	141 (47.6)	84 (28.4)	41 (13.9)	
	Physicians, n (%)	36 (8.4)	165 (38.6)	158 (36.9)	69 (16.1)	

**Fig. 1:**
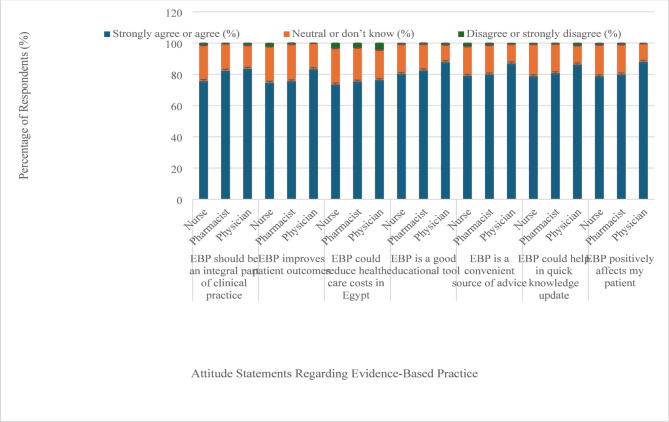
Perceptions of evidence-based practice among physicians, pharmacists, and nurses

Figure 1 below illustrates HCPs attitudes toward EBP across several key parameters. Physicians generally showed higher agreement with EBP principles than nurses and pharmacists, particularly in areas such as its integration into clinical practice and its potential to improve patient outcomes. A strong majority of all professions agreed that EBP should be a central component of clinical practice, with 83.5% of physicians, 82.3% of pharmacists, and 75.7% of nurses expressing this view. Additionally, most HCPs recognized EBP’s role in enhancing patient outcomes, with 83.3% of physicians, 75.5% of pharmacists, and 74.6% of nurses affirming its effectiveness. All groups also strongly agreed on EBP’s value as an educational tool and a resource for updating clinical knowledge. Regarding its impact on healthcare costs and its ability to quickly update knowledge, there was broad consensus among HCPs. Notably, a significant proportion of all professions indicated interest in attending workshops or courses on EBP, highlighting a willingness to further engage with and improve their practice in this area.

Table 4 highlights the varying frequencies with which nurses, pharmacists, and physicians engage in evidence-based practices. The post hoc analysis showed significant differences in the frequency of evidence-based practice (EBP) activities across healthcare professions. Pharmacists and physicians were significantly more likely than nurses to search electronic databases daily (*p* < 0.001 for both comparisons), while physicians also searched more frequently than pharmacists (*p* = 0.030). Similarly, physicians read clinical research reports daily more often than both pharmacists (*p* < 0.001) and nurses (*p* < 0.001), with no significant difference between pharmacists and nurses (*p* = 0.476).

For discussing research findings (e.g., journal clubs), physicians did so more frequently than pharmacists (*p* < 0.001) and nurses (*p* < 0.001), while no significant difference was found between pharmacists and nurses (*p* = 0.347). Physicians also relied on clinical experience more consistently than pharmacists and nurses (*p* < 0.001 for both comparisons), with no difference between pharmacists and nurses (*p* = 0.293).

Regarding the use of clinical practice guidelines, physicians reported using them more often than both pharmacists (*p* < 0.001) and nurses (*p* = 0.001), while pharmacists were more likely to use them than nurses (*p* = 0.001). Similarly, physicians accessed online databases (e.g., MEDLINE, Cochrane) more frequently than pharmacists (*p* = 0.002) and nurses (*p* < 0.001). Physicians also made more use of medical websites (e.g., UpToDate, Medscape) than both pharmacists (*p* < 0.001) and nurses (*p* < 0.001), with pharmacists using them more frequently than nurses (*p* = 0.001).

In summary, physicians consistently demonstrated higher engagement in EBP activities, followed by pharmacists, while nurses showed the lowest frequency of participation across various EBP-related tasks.

**Table 4:** Frequency and distribution of Evidence-Based Practice-Related activities among HCPs

Parameter		Profession			*P* value	
In the last year, how often have you		Nurse *N* = 196	Pharmacist *N* = 395	Physician *N* = 313		
**Searched an electronic database**	Daily, n (%)	11 (5.6)	88 (22.3)	50 (16.0)		0.00*
	Weekly, n (%)	38 (19.4)	81 (20.5)	88 (28.1)		
	Twice a month, n (%)	28 (14.3)	50 (12.7)	41 (13.1)		
	Monthly or less, n (%)	52 (26.5)	80 (20.3)	77 (24.6)		
	Never or N/A, n (%)	67 (34.2)	96 (24.3)	57 (18.2)		
**Read published clinical research reports**	Daily, n (%)	4 (2.0)	13 (3.3)	10 (3.2)		0.00*
	Weekly, n (%)	17 (8.7)	44 (11.1)	72 (23.0)		
	Twice a month, n (%)	17 (8.7)	38 (9.6)	48 (15.3)		
	Monthly or less, n (%)	71 (36.2)	158 (40.0)	136 (43.5)		
	Never or N/A, n (%)	87 (44.4)	142 (35.9)	47 (15.0)		
**Discussed literature or research finding with others/practice (e.g.**,** journal club)**	Daily, n (%)	3 (1.5)	1 (0.3)	3 (1.0)		0.00*
	Weekly, n (%)	5 (2.6)	15 (3.8)	23 (7.3)		
	Twice a month, n (%)	11 (5.6)	16 (4.1)	27 (8.6)		
	Monthly or less, n (%)	41 (20.9)	97 (24.6)	113 (36.1)		
	Never or N/A, n (%)	136 (69.4)	266 (67.3)	147 (47.0)		
**How often do you use the following information sources in guiding your clinical decisions?**						
**Personal clinical experience**	Always, n (%)	45 (23.0)	70 (17.7)	108 (34.5)		0.00*
	Most of time/often, n (%)	63 (32.1)	112 (28.4)	119 (38.0)		
	Sometimes, n (%)	57 (29.1)	121 (30.6)	70 (22.4)		
	Rarely, n (%)	9 (4.6)	28 (7.1)	10 (3.2)		
	Never or N/A, n (%)	22 (11.22)	64 (16.20)	6 (1.9)		
**Journal articles**	Always, n (%)	18 (9.2)	26 (6.6)	35 (11.2)		0.00*
	Most of time/often, n (%)	44 (22.4)	77 (19.5)	97 (31.0)		
	Sometimes, n (%)	72 (36.7)	146 (37.0)	144 (46.0)		
	Rarely, n (%)	30 (15.3)	65 (16.5)	26 (8.3)		
	Never or N/A, n (%)	16 (8.2)	33 (8.4)	4 (1.3)		
**Opinion of colleagues**	Always, n (%)	24 (12.2)	29 (7.3)	42 (13.4)		0.00*
	Most of time/often, n (%)	57 (29.1)	101 (25.6)	111 (35.5)		
	Sometimes, n (%)	81 (41.3)	175 (44.3)	136 (43.5)		
	Rarely, n (%)	17 (8.7)	47 (11.9)	21 (6.7)		
	Never or N/A, n (%)	17 (8.67)	43 (10.8)	3 (0.9)		
**Clinical practice guidelines**	Always, n (%)	32 (16.3)	93 (23.5)	73 (23.3)		0.00*
	Most of time/often, n (%)	60 (30.6)	92 (23.3)	125 (39.9)		
	Sometimes, n (%)	68 (34.7)	90 (22.8)	86 (27.5)		
	Rarely, n (%)	10 (5.1)	38 (9.6)	16 (5.1)		
	Never or N/A, n (%)	26 (13.26)	82 (20.8)	13 (4.15)		
**Online Database (e.g.**,** Medline**,** Cochrane**,** TRIP database).**	Always, n (%)	30 (15.3)	56 (14.2)	43 (13.7)		0.00*
	Most of time/often, n (%)	45 (23.0)	103 (26.1)	86 (27.5)		
	Sometimes, n (%)	56 (28.6)	120 (30.4)	125 (39.5)		
	Rarely, n (%)	20 (10.2)	47 (11.9)	36 (11.5)		
	Never or N/A, n (%)	45 (23.07)	69 (17.5)	23 (7.35)		
**Medical websites (e.g.**,** e-medicine**,** UpToDate**,** Medscape)**	Always, n (%)	41 (20.9)	138 (34.9)	82 (26.2)		0.00*
	Most of time/often, n (%)	57 (29.1)	116 (29.4)	124 (39.6)		
	Sometimes, n (%)	57 (29.1)	91 (23.0)	91 (29.1)		
	Rarely, n (%)	12 (6.1)	17 (4.3)	13 (4.2)		
	Never or N/A, n (%)	29 (14.8)	33 (8.35)	3 (0.96)		

The utilization patterns of various medical databases among nurses, pharmacists, and physicians revealed notable differences, as summarized in Table 5. Significant differences (p-value < 0.05) were observed in the frequencies of responses across the professional groups. For the response category “Have read it but not used in clinical decision-making,” pharmacists and physicians reported similar percentages (18.0% and 18.8%, respectively), which were significantly higher than nurses (12.1%). In contrast, the category “Read and used in clinical decision-making” demonstrated marked differences, with physicians consistently reporting the highest usage of evidence-based resources such as PubMed (44.4%), UpToDate (39.0%), and Lexicomp (11.2%). Pharmacists showed intermediate levels of usage, with percentages ranging from 30.4% (UpToDate) to 34.9% (Lexicomp), while nurses reported significantly lower usage across all resources.

EBP database awareness also varied significantly. A substantial proportion of nurses were unaware of key databases, including PubMed (37.2%), UpToDate (35.7%), and Lexicomp (68.9%). Pharmacists exhibited greater awareness than nurses but less than physicians, who demonstrated the highest levels of familiarity and usage of these databases. The significant p-values indicate that the observed differences in usage and awareness of medical databases across the three professional groups are unlikely to be due to chance and reflect practical differences in how these resources are integrated into their clinical practice (Table 5).

Additionally, over 70% of healthcare professionals across all professions were unfamiliar with other evidence-based resources, including TRIP, while more than 60% were unaware of Dynamed Plus and Ovid Medline. Furthermore, more than 50% did not recognize resources like Evidence Alerts, ACP, CEBM, DARE, and Cochrane, indicating a broad lack of familiarity with these evidence-based practice tools.

**Table 5:** Utilization of medical databases by nurses, pharmacists, and physicians in terms of frequencies and percentages

Parameter		Profession			P-value*
		Nurse *N* = 196	Pharmacist *N* = 395	Physician *N* = 313	
PubMed, Embase, Science Index or CINAHL	Have read it but not used in clinical decision making, n (%)	25 (12.1)	71 (18.0)	59 (18.8)	0.00*
	Heard about it, n (%)	62 (31.6)	84 (21.3)	74 (23.6)	
	Read and used in clinical decision making, n (%)	36 (18.4)	122 (30.9)	139 (44.4)	
	Unaware, n (%)	73 (37.2)	118 (29.9)	41 (13.1)	
UpToDate	Have read it but not used in clinical decision making, n (%)	22 (11.2)	39 (9.9)	51 (16.3)	0.00*
	Heard about it, n (%)	74 (37.8)	113 (28.6)	89 (28.4)	
	Read and used in clinical decision making, n (%)	30 (15.3)	120 (30.4)	122 (39.0)	
	Unaware, n (%)	70 (35.7)	123 (31.1)	51 (16.3)	
Lexicomp	Have read it but not used in clinical decision making, n (%)	17 (8.7)	41 (10.4)	30 (9.6)	0.00*
	Heard about it, n (%)	38 (19.4)	72 (18.2)	48 (15.3)	
	Read and used in clinical decision making, n (%)	6 (3.1)	138 (34.9)	35 (11.2)	
	Unaware, n (%)	135 (68.9)	144 (36.5)	200 (63.9)	
eMedicine	Have read it but not used in clinical decision making, n (%)	25 (12.8)	35 (8.9)	48 (15.3)	0.00*
	Heard about it, n (%)	58 (29.6)	131 (33.2)	110 (35.1)	
	Read and used in clinical decision making, n (%)	17 (8.7)	39 (9.9)	78 (24.9)	
	Unaware, n (%)	96 (49.0)	190 (48.1)	77 (24.6)	
BMJ Evidence-Based Medicine	Have read it but not used in clinical decision making, n (%)	19 (9.7)	30 (7.6)	43 (13.7)	0.00*
	Heard about it	46 (23.5)	74 (18.7)	90 (28.8)	
	Read and used in clinical decision making, n (%)	7 (3.6)	51 (12.9)	61 (19.5)	
	Unaware	124 (63.3)	240 (60.8)	119 (38.0)	

*Significance difference, Chi square test is used

Figure 1: Percentage of healthcare professionals rating various barriers to evidence-based practice (EBP) as a “Major Problem”

**Fig. 2:**
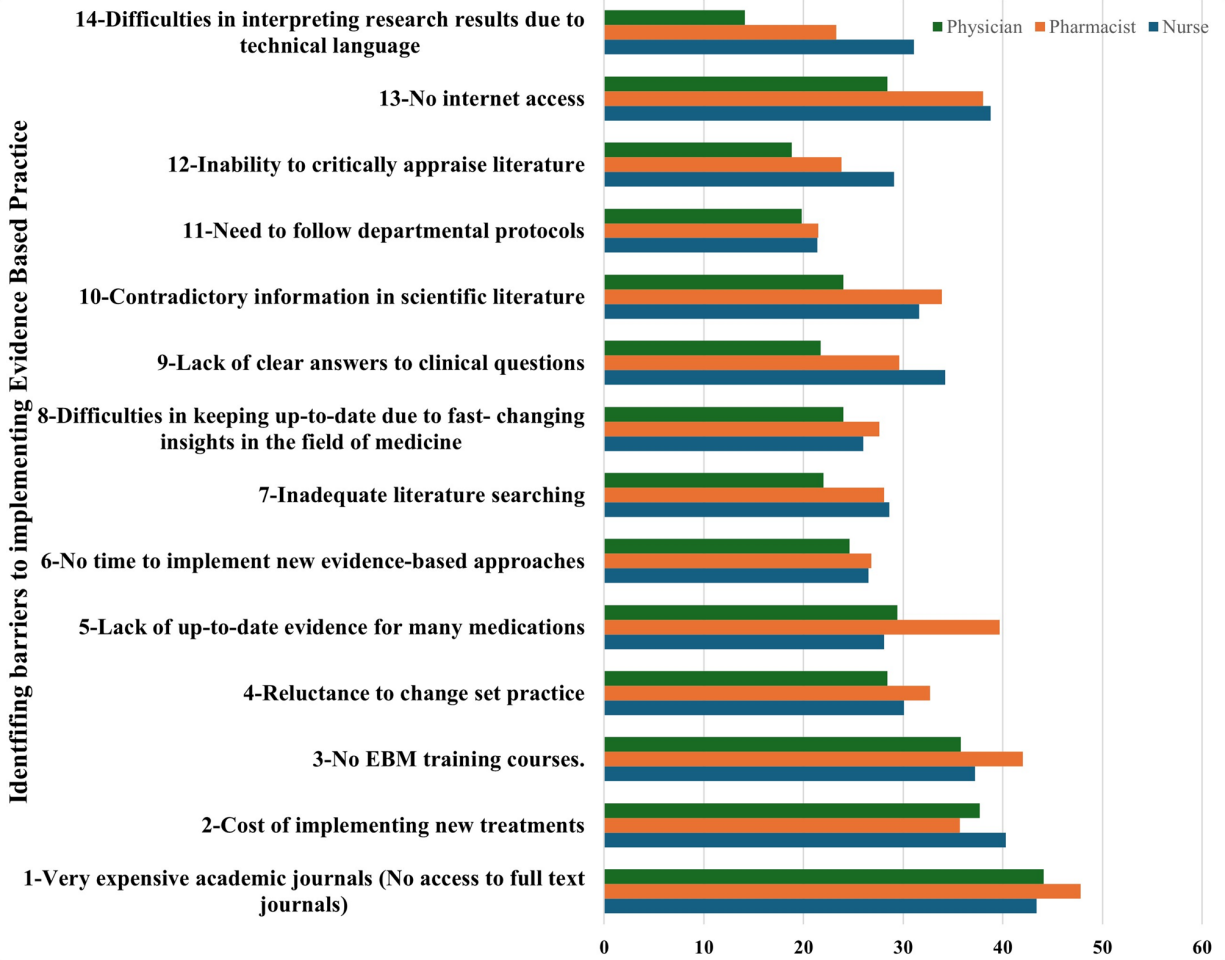
Percentage of HCPs rating various barriers to EBP as a “Major Problem”

Our survey addressed several barriers to evidence-based practice, asking HCPs to rate these barriers on a scale from 1 (“no problem”) to 5 (“major problem”). Figure 2 below shows the percentage of healthcare professionals who rated these barriers as a “major problem” [5].

As illustrated in Fig. 2, The lack of EBP training courses was notably identified as a key issue, with a large percentage of professionals rating it as a “major problem” (rating 5). Specifically, 37.2% of nurses, 42.0% of pharmacists, and 35.8% of physicians indicated this as a significant barrier. Another substantial challenge was the inaccessibility to full-text academic journals due to high cost, with 43.4% of nurses, 47.8% of pharmacists, and 44.1% of physicians rating this as a major issue. Additionally, the cost of implementing new treatments was reported as a barrier, with 40.3% of nurses, 35.7% of pharmacists, and 37.7% of physicians rating this problem at the highest level.

**Table 6:** HCPs’ opinions regarding some of the suggested ways to move towards evidence-based practice are expressed in frequency and percentage

Option	Response	Profession			P value
		Nurse *N* = 196	Pharmacist *N* = 395	Physician *N* = 313	
Using evidence based Practice guidelines & Protocols	Method currently used, n (%)	55 (28.1)	101 (25.6)	80 (25.6)	0.274
	Method of potential interest for future use, n (%)	116 (59.2)	239 (60.5)	205 (65.5)	
	Not interesting/Not applicable in my area of expertise, n (%)	25 (12.8)	55 (13.9)	28 (8.9)	
Dedicating time during the working hours for research activities	Method currently used, n (%)	38 (19.4)	65 (16.5)	48 (15.3)	0.003*
	Method of potential interest for future use, n (%)	121 (61.7)	257 (65.1)	235 (75.1)	
	Not interesting/Not applicable in my area of expertise, n (%)	37 (18.9)	73 (18.5)	30 (9.6)	
Attending educational sessions on the utilization of research findings	Method currently used, n (%)	54 (27.6)	62 (15.7)	64 (20.4)	0.002*
	Method of potential interest for future use, n (%)	113 (57.7)	284 (71.9)	220 (70.3)	
	Not interesting/Not applicable in my area of expertise, n (%)	29 (14.8)	49 (12.4)	29 (9.3)	
The availability of research and development department	Method currently used, n (%)	54 (27.6)	83 (21.0)	73 (23.3)	0.054
	Method of potential interest for future use, n (%)	117 (59.7)	260 (65.8)	216 (69.0)	
	Not interesting/Not applicable in my area of expertise, n (%)	25 (12.8)	52 (13.2)	24 (7.7)	

*Significance difference, Chi square test is used

The results in Table 6 above indicate HCPs’ opinions on ways to advance EBP. The post hoc analysis of healthcare professionals’ opinions revealed significant differences across professions in some areas. For dedicating time during working hours for research activities, nurses reported significantly higher current practice (19.4%) compared to pharmacists (16.5%) and physicians (15.3%) (P = 0.003). However, considerable interest in this practice for future use was observed across all professions, with physicians indicating the highest interest (75.1%), followed by pharmacists (65.1%) and nurses (61.7%). Regarding attending educational sessions on utilizing research findings, nurses (27.6%) were significantly more likely to participate compared to pharmacists (15.7%) and physicians (20.4%) (P = 0.002). Future interest in this method was high among all groups, with no significant differences observed. For the availability of research and development departments, no significant differences were found among the groups for current usage (P = 0.054), although interest in future use remained high across all professions. For using evidence-based practice guidelines and protocols, no significant differences were observed in current usage (P = 0.274). Physicians reported the lowest percentage of respondents indicating this approach as ‘not interesting or not applicable’ (8.9%), suggesting broader relevance for this group compared to nurses (12.8%) and pharmacists (13.9%).

## Discussion

This descriptive cross-sectional study explored KAP level of EBP among 1,396 HCPs, comprising pharmacists, nurses, and physicians, with notable differences across demographic and professional characteristics. HCPs holding at least a bachelor’s degree, who are in daily contact with other healthcare professionals and patients, and are currently part of the workforce, were invited to complete the questionnaire via SurveyMonkey. The platform allows only one attempt per email, ensuring no duplication of responses. To our knowledge, this is the first cross-sectional study evaluating knowledge, attitude, and EBP among physicians, nurses, and pharmacists in Egypt. In contrast to many earlier studies, which were often focused on a single profession and setting, this survey gathered many respondents from a variety of practice settings, specialties, and healthcare professions, giving a better picture of the general knowledge and application of EBP in Egypt.

In our study, the majority of pharmacist and nurse respondents were female, whereas most physician respondents were male. This could be attributed to the lengthy career pathway, societal pressures to prioritize family responsibilities, demanding work hours, frequent on-call duties—particularly in specialties like intensive care and emergency medicine and the extensive qualifications required to become a physician [26, 27].

Nurses were the youngest group while physicians were the oldest. This can be attributed to the shorter duration of formal education required for their profession. This allows nurses to enter the workforce at a younger age. They can begin their careers with bachelor’s degrees which take significantly less time to complete compared to the lengthy educational and residency requirements for physicians.

In contrast, physicians typically spend over a decade completing their education, including undergraduate studies, medical school, internships, and residencies, which delays their entry into the workforce. Additionally, nursing tends to have higher rates of career turnover or attrition due to factors such as burnout, the physical demands of the job, job dissatisfaction, and long shifts [28, 29]. This turnover often results in younger nurses entering the field to fill workforce gaps, lowering the average age of nurses.

Physicians tended to have more years of experience compared to other groups. This is due to their extended period of training, which often includes postgraduate specialization, fellowships, or sub specializations. This also explains why physicians have the highest percentage of postgraduate qualifications compared to other HCPS.

The current study findings identified most participants were aware of EBP with physicians (81.31%) showing the highest awareness, followed by nurses (66.89%) and pharmacists (56.85). However, fewer have actively participated in courses or workshops on the subject. This is consistent with a previous KAP study on EBM, which reported that while 94.7% of Egyptian physicians were familiar with the term EBM, only 37.3% had attended prior EBM training [13]. Similarly, a study conducted in Ethiopia among 115 healthcare professionals found that although 72% (83 participants) were aware of EBM, only 35% had attended a course or workshop on the subject [30].

On the same hand, participation in EBM-related courses was low across all groups, with physicians showing highest overall participation. This is possibly because physicians need post-graduate degrees for specialization, which often incorporates EBM principles. This is clearly reflected in the study sample where 64% of the physicians had a post-graduate degree, compared to 40% of the pharmacists and 28% of the nurses.

Physicians demonstrated superior understanding of key EBM terms such as ‘relative risk,’ ‘meta-analysis,’ and ‘RCTs’ compared to nurses and pharmacists, while no significant differences were observed for ‘confidence intervals.’ This finding aligns with the Saudi Arabian study, which reported variability in physicians’ understanding of statistical terms, with better comprehension of ‘relative risk’ and ‘absolute risk’ but lower understanding of ‘confidence intervals’ [19]. Similarly, another study from Egypt revealed that 48.7% of physicians understood ‘case-control studies’ well enough to explain them, followed by ‘randomized controlled trials’ (44.7%), ‘relative risk’ (40.7%), and ‘absolute risk’ (34.7%). However, understanding of ‘confidence intervals’ was lower, with only 24% able to explain the concept [13]. However, it contrasts with the Syrian study, which showed an overall low level of understanding of statistical terms, including ‘relative risk,’ ‘absolute risk,’ and ‘meta-analysis,’ among physicians.” [31].

The findings of this study also revealed that most of respondents in our survey showed a positive attitude towards EBP and believed that EBP improves patient outcomes, decreases healthcare costs in Egypt, and helps update knowledge more rapidly between different professionals. In addition, all groups supported integrating EBP into clinical practice, with physicians showing the strongest agreement (83.5%). Similar conclusions were drawn in earlier studies, such as the assessment at the National Liver Institute, which reported that most physicians held favorable attitudes toward EBP and acknowledged its role in enhancing clinical decision-making and patient care [13]. Likewise, Egyptian family physicians expressed strong beliefs in EBM’s benefits for clinical practice and healthcare outcomes [32]. Nationwide surveys in Taiwan among multidisciplinary teams, including nurses, pharmacists, and allied health professionals, further highlighted positive attitudes toward EBP, though barriers such as insufficient resources and training were noted [6]. Similarly, pharmacists acknowledged the utility of EBP but identified challenges like lack of time and training [33], while Egyptian nurses expressed generally supportive beliefs despite facing hurdles in EBP implementation [34].

Physicians were the most active in using EBP resources, such as PubMed and UpToDate, followed by pharmacists and nurses. This aligns with findings from Taiwan, where physicians were more skilled in conducting EBP and applying it in clinical decision-making compared to other professionals [6].

One study has revealed that EBP among Egyptian nurses was low because of the English language barriers and that Egyptian nurses rely on their personal experience to provide patient care rather than EBP [34]. Nonetheless, another study reported that Egyptian nurses highly agreed that they can search for the best evidence to answer a question however, there is no time for internet search during work [35]. These findings in earlier studies could explain why nurses have shown the least engagement with EBP resources.

The present study identified key barriers to the implementation of EBP among healthcare professionals, including the lack of EBP training courses, limited access to full-text academic journals due to high cost, and the expenses associated with implementing new treatments. Similar barriers have been reported in other studies. For instance, a study in Ethiopia highlighted a lack of EBP training and difficulties in accessing resources, such as internet and time constraints, as major challenges [36]. Similarly, a study conducted in Iran identified organizational barriers such as a shortage of nurses (78.3%), lack of internet access at the workplace (72.2%), and heavy workloads (70.0%) as significant challenges. On an individual level, the most prominent barriers included a lack of time to read literature (83.7%) and insufficient ability to work with computers, which hindered the implementation of evidence-based practice [37].

HCPs in this study were highly interested in dedicating time during working hours for research activities and attending educational sessions on utilizing research findings to implement EBP in the future. Lack of time and scarce communication between academic and clinical practice environments were the most common barriers reported in many studies [13, 34, 38–41].

The observed differences in EBP engagement among physicians, pharmacists, and nurses can be interpreted in light of their varying roles in clinical decision-making. Physicians typically serve as the primary decision-makers in patient diagnosis and treatment, which necessitates a stronger reliance on up-to-date evidence to guide practice. Pharmacists, particularly those in clinical settings, increasingly participate in therapeutic decision-making by offering evidence-based recommendations on drug therapy. Nurses, while critical to patient care and implementation of treatment plans, may have fewer opportunities to initiate therapeutic decisions, potentially influencing their reported use of EBP. These findings are consistent with previous studies that highlight how professional roles and scope of practice shape the integration of EBP in daily clinical activities [42, 43]. Nonetheless, promoting EBP across all healthcare professions remains essential to improving overall care quality and patient outcomes.

These results highlight significant differences in EBP awareness, training, and practice among HCPs, emphasizing the need for tailored interventions to enhance EBP adoption across professions.

### Recommendations

Given the evident gaps in EBP training and awareness among healthcare professionals in Egypt, several recommendations can be made. First, targeted educational initiatives should be introduced across all healthcare disciplines, particularly for nurses and pharmacists, to improve EBP competencies such as critical appraisal and biostatistics. These could include integrating EBP modules into undergraduate and postgraduate curricula, as well as offering continuing professional development (CPD) workshops.

Second, healthcare institutions should prioritize creating a culture that supports EBP through administrative support, access to digital resources, and protected time for research and evidence consultation. Appointing EBP champions or mentors within clinical departments could facilitate practice change and peer learning.

Third, at the policy level, the Ministry of Health and educational accrediting bodies should consider mandating EBP training as part of licensing or re-certification processes. National guidelines promoting EBP use in clinical decision-making would help standardize practices across facilities.

Finally, future research should explore the barriers to EBP adoption in specific healthcare settings (e.g., rural vs. urban, public vs. private) and test the effectiveness of targeted interventions in improving EBP knowledge and uptake.

### Limitations

The main limitation of this study is that it relied on a web-based survey, which may have restricted access to certain populations, particularly healthcare professionals with limited internet access or low digital literacy. The cross-sectional design also restricts our ability to make causal inferences about the relationships observed. Finally, self-reported responses may introduce social desirability bias, potentially affecting the accuracy of the data.

### Theoretical implications

This study contributes to the growing body of literature on evidence-based practice (EBP) readiness by providing empirical data from a Middle Eastern context, specifically Egypt, which is underrepresented in existing research. The findings support existing theories that emphasize the role of professional roles, institutional support, and prior exposure to EBP in shaping readiness. Our results also highlight the need for culturally and contextually adapted EBP frameworks that account for local healthcare infrastructure and professional norms.

### Practical implications

The findings of this study suggest a clear need for structured EBP training programs targeted at different healthcare professions, especially nurses and pharmacists who reported lower readiness scores. Healthcare institutions should invest in continuous professional development programs that include EBP as a core component. Additionally, policy makers should consider integrating EBP competencies into national licensing requirements and healthcare curricula to strengthen decision-making capacity and patient outcomes across the system.

## Conclusion

This study identifies gaps in of knowledge, attitudes, and practices related to EBP among pharmacists, nurses, and physicians in Egypt. Physicians demonstrated the highest levels of knowledge, engagement, and understanding of EBP concepts, followed by pharmacists, with nurses showing the least engagement and understanding. While awareness of the EBP term was high across all groups, participation in EBM-related training and activities remained limited, particularly among nurses. Despite the limited EBP training, HCPs across all specialties showed a positive attitude towards EBP, recognizing its significant role in enhancing clinical practice, patient outcomes, and education.

## Data Availability

The datasets generated during and/or analyzed during the current study are available from the corresponding author upon reasonable request.

## Declarations

### Clinical trial registration

Clinical trial number: Not applicable.

## Abbreviations

EBP: Evidence based practice
RCT: Randomized controlled trials
KAP: Knowledge, attitude, and practice
HCP: Healthcare professionals
EBM: Evidence based medicine
SPSS: Statistical packages for social science
N: Number
BMJ: British medical journal
